# Supplementary material for: Using qualitative research methods to understand how surgical procedures and devices are introduced into NHS hospitals: the Lotus study protocol
Source: BMJ Open. 2021 Dec 3;11(12):e049234. doi: 10.1136/bmjopen-2021-049234 (PMC8647399; doi:10.1136/bmjopen-2021-049234)
Supplement: Author's manuscript [file bmjopen-2021-049234.draft_revisions.pdf]

BMJ Open is committed to open peer review. As part of this commitment we make the peer review history of every article we publish publicly available.

When an article is published we post the peer reviewers' comments and the authors' responses online. We also post the versions of the paper that were used during peer review. These are the versions that the peer review comments apply to.

The versions of the paper that follow are the versions that were submitted during the peer review process. They are not the versions of record or the final published versions. They should not be cited or distributed as the published version of this manuscript.

BMJ Open is an open access journal and the full, final, typeset and author-corrected version of record of the manuscript is available on our site with no access controls, subscription charges or pay-per-view fees (http://bmjopen.bmj.com).

If you have any questions on BMJ Open's open peer review process please email info.bmjopen@bmj.com

BMJ Open

Understanding how surgical procedures and devices are introduced into NHS hospitals: The Lotus study protocol

Journal:	*BMJ Open*
Manuscript ID	bmjopen-2021-049234
Article Type:	Protocol
Date Submitted by the Author:	19-Jan-2021
Complete List of Authors:	Elliott, Daisy; University of Bristol<br>Blencowe, Natalie; University of Bristol, Centre for Surgical Research<br>Cousins, Sian; University of Bristol, Population Health Sciences<br>Zahra, Jesmond; University of Bristol, Centre for Surgical Research<br>Skilton, Anni; University of Bristol<br>Mathews, Johnny; University of Bristol, Population Health Sciences<br>Paramasivan, Sangeetha ; University of Bristol<br>Hoffmann, Christin; University of Bristol<br>McNair, Angus; University of Bristol, Centre for Surgical Research, Bristol Medical School: Population Health Sciences; North Bristol NHS Trust, GI Surgery<br>Ochieng, Cynthia; University of Bristol<br>Richards, Hollie; University of Bristol, Population Health Sciences<br>Hossaini, Sina; University of Bristol<br>Scroggie, Darren; University of Bristol<br>Main, Barry; University of Bristol, School of Social and Community Medicine<br>Potter, Shelley; University of Bristol<br>Avery, Kerry; University of Bristol<br>Donovan, Jenny; University of Bristol<br>Blazeby, Jane; University of Bristol
Keywords:	SURGERY, QUALITATIVE RESEARCH, Quality in health care < HEALTH SERVICES ADMINISTRATION & MANAGEMENT

SCHOLARONE™
Manuscripts

I, the Submitting Author has the right to grant and does grant on behalf of all authors of the Work (as defined in the below author licence), an exclusive licence and/or a non-exclusive licence for contributions from authors who are: i) UK Crown employees; ii) where BMJ has agreed a CC-BY licence shall apply, and/or iii) in accordance with the terms applicable for US Federal Government officers or employees acting as part of their official duties; on a worldwide, perpetual, irrevocable, royalty-free basis to BMJ Publishing Group Ltd ("BMJ") its licensees and where the relevant Journal is co-owned by BMJ to the co-owners of the Journal, to publish the Work in this journal and any other BMJ products and to exploit all rights, as set out in our licence.

The Submitting Author accepts and understands that any supply made under these terms is made by BMJ to the Submitting Author unless you are acting as an employee on behalf of your employer or a postgraduate student of an affiliated institution which is paying any applicable article publishing charge ("APC") for Open Access articles. Where the Submitting Author wishes to make the Work available on an Open Access basis (and intends to pay the relevant APC), the terms of reuse of such Open Access shall be governed by a Creative Commons licence – details of these licences and which Creative Commons licence will apply to this Work are set out in our licence referred to above.

Other than as permitted in any relevant BMJ Author's Self Archiving Policies, I confirm this Work has not been accepted for publication elsewhere, is not being considered for publication elsewhere and does not duplicate material already published. I confirm all authors consent to publication of this Work and authorise the granting of this licence.

For peer review only

Understanding how surgical procedures and devices are introduced into NHS hospitals: The Lotus study protocol

Submission category: Protocol

Authors: Elliott D[1,], Blencowe NS[1, 2], Cousins S[1], Zahra J[1], Skilton A[1], Mathews JA[1], Paramasivan S[1], Hoffmann C[1], McNair AGK[1,3], Ochieng C[1], Richards H[1], Hossaini S[1], Scroggie D[1], Main BG[1,2], Potter S[1], Avery KNL[1], Donovan JL [4], Blazeby JM[1, 2]

Affiliations:

[1]National Institute for Health Research Bristol Biomedical Research Centre, Surgical Innovation Theme, Centre for Surgical Research, University of Bristol, 39 Whatley Road, Clifton, Bristol, UK, BS8 2PS

[2] University Hospitals Bristol and Weston NHS Foundation Trust, Bristol, UK, BS2 8HW

[3] North Bristol NHS Trust, Bristol, UK, BS10 5NB

[4] Population Health Sciences, Bristol Medical School, University of Bristol, 39 Whatley Road, Clifton, Bristol, UK, BS8 2PS

Correspondence to: Daisy Elliott [daisy.elliott@bristol.ac.uk]

Running title: LOTUS STUDY PROTOCOL

Keywords: Surgery; Innovation; Qualitative

Funding statement: This study was supported by the NIHR Biomedical Research Centre at University Hospitals Bristol and Weston NHS Foundation Trust and the University of Bristol. The views expressed in this publication are those of the author(s) and not necessarily those of the NHS, the National Institute for Health Research or the Department of Health and Social Care. JLD and JMB are NIHR Senior Investigators. NSB and AGKM are MRC Clinician Scientists.

Word count: 1908 (excluding title page, abstract, tables and references)

For peer review only - http://bmjopen.bmj.com/site/about/guidelines.xhtml

Abstract

Introduction:

The development of innovative invasive procedures and devices are essential to improving outcomes in healthcare. However, how these are introduced into practice has not been studied in detail. The Lotus study will follow a wide range of 'case studies' of new procedures and/or devices being introduced into NHS trusts to explore what information is communicated to patients, how procedures are modified over time, and how outcomes are selected and reported.

Methods and analysis:

This qualitative study will use ethnographic approaches to investigate how new invasive procedures and/or devices are introduced. Consultations in which the innovation is discussed will be audio-recorded to understand information provision practice. To understand if and how procedures evolve, they will be video recorded and non-participant observations will be conducted. Post-operative interviews will be conducted with the innovating team and patients who are eligible for the intervention. Audio recordings will be audio-recorded, transcribed verbatim and analysed thematically using constant comparison techniques. Video recordings will be reviewed to deconstruct procedures into key components and document how the procedure evolves. Comparisons will be made between the different data sources.

Ethics and dissemination:

The study protocol has HRA and Health and Care Research Wales approval (Ref 18/SW/0277). Results will be disseminated at appropriate conferences and will be published in peer-reviewed journals. The findings of this study will provide a better understanding of how innovative invasive procedures and/or devices are introduced into practice.

(233 words)

Strengths and limitations of this study

- Studying innovation in real-time is novel and enables in-depth and contemporaneous insights
- Including a range of specialities and hospitals will provide a broad overview of how innovative invasive procedures and devices are introduced into NHS hospitals
- Triangulation of multiple methods of data collection will facilitate a comprehensive understanding of surgical innovation and help to validate findings

For peer review only

Introduction

At least 12.5 million invasive procedures are delivered in the UK annually[1]. An innovative invasive procedure can be defined as a new or modified procedure that differs from currently accepted local practice, the outcomes of which have not been fully systematically evaluated and reported in a standardised manner, and which may entail unknown risks to the patient[2 3]. This can include the introduction of entirely new procedures as well as undertaking modifications to existing techniques, with the latter being more common [4]. Innovation is critical and constantly occurring because of the drive to improve a range of outcomes. Common targets include using less invasive techniques; shortening hospital stays; improving patient outcomes; or creating novel therapeutic options[4-6]. Innovation also frequently occurs because of a healthcare professional's desire to pioneer new techniques and to be recognised as a leader in the field [4]. An independent review by the Royal College of Surgeons of England has predicted that surgery will undergo a massive transformation because of developments in new technologies such as robotics, artificial intelligence, genomics and digital technologies, and concluded that the world of surgery is embarking on a time of innovation[7].

Unlike the pharmaceutical industry, where there are extensive regulatory and ethical requirements[8-10], the introduction of innovative invasive procedures and/or devices (IP/Ds) is not adequately regulated. The IDEAL framework [11] outlines five consecutive stages of development, evaluation and dissemination of innovative IP/Ds, although its uptake has been slow [12 13] and it is often incorrectly followed [14]. Furthermore, whilst innovative IP/Ds should be introduced in the context of formal research studies or in accordance with local hospital policies and processes[15], ethical approval is rarely gained [10] and procedures can be introduced without any formal governance from local hospitals [16 17]. Important questions have also been raised surrounding informed consent for innovative IP/Ds. For instance, an inquest into the death of the first patient in the UK to undergo robotically assisted heart surgery found the patient had not been fully informed about the comparative risks of robotic versus conventional open surgery[16].

A recent independent review has highlighted the need for better governance and evaluation of surgical innovation [18]. As little is known about mechanisms of how new IP/Ds are introduced into practice[19], a greater understanding of how innovative procedures are introduced is imperative to developing guidance and regulation. Complex phenomena such as innovative IP/Ds can be explored in-depth using qualitative methodology so that rich insights and experiences of innovation from a number of perspectives can be captured and explored in detail within their original setting[20 21]. The Lotus study will use qualitative research methods to provide in-depth, real-time insights to how

For peer review only - http://bmjopen.bmj.com/site/about/guidelines.xhtml

innovative IP/Ds are introduced into NHS hospitals. Specific objectives of the Lotus study are to explore: i) how information is communicated to patients and how patients understand this information in order to inform their decision-making process; ii) how procedures evolve over time; and iii) how outcomes are selected, measured and reported.

Methods and analysis

Design

The Lotus study will use ethnographic approaches to investigate how new invasive IP/Ds are introduced. A grounded theory methodology will enable the inductive identification of themes that are derived or grounded in the data [22 23]. Its central principle is of constant comparison, where new findings are systematically compared with existing data so that similarities and differences can be identified through the ongoing assimilation of data [22 24].

Case study eligibility

Case studies will follow the introduction of innovative IP/Ds in the NHS. An invasive procedure will be defined as one in which purposeful and deliberate access to the body is gained via an incision or percutaneous puncture, instrumentation is used in addition to a puncture needle, or instrumentation occurs via a natural orifice. It begins when entry to the body is gained and ends when the instrument is removed and/or the skin is closed. It is performed by trained healthcare professionals using instruments, which include, but are not limited to, endoscopes, catheters, scalpels, scissors, devices and tubes[25]. A device is defined as any instrument, apparatus, appliance, software, material or other article, whether used alone or in combination, which is intended by the manufacturer to be used for human beings[26].

A senior academic surgeon and the BRC Surgical Innovation theme lead (JMB) will approach individuals from NHS trusts, regulators (e.g. NHS New Procedure Committees and ethics committees) and funding bodies to identify innovative IP/Ds. Potential case studies will be screened by the research team. Firstly, an in-depth qualitative interview with the innovator (a 'background' interview) will be conducted to learn more about the innovative IP/D. Alongside this, the team will undertake a literature review to identify existing published articles on the procedure. A data extraction form will be developed specifically for the review to record details of each article (relating to authors, year, country of origin, study design, participants, and number of participating centres). We will aim to follow a range of case studies to capture innovation in different contexts, including varying stages of innovation (as identified by the IDEAL framework[11]), type of innovation (procedure

For peer review only - http://bmjopen.bmj.com/site/about/guidelines.xhtml

or device), surgical specialty, geographical location, and trust type. Where possible, we will try to identify innovative IP/Ds that are due to be introduced into practice to enable us to capture data from the first time they are performed. Identification of new case studies will continue until additional data are not adding anything new to the analytical framework and saturation is felt to have been achieved.

Recruitment and sampling

For each case study, participants will include healthcare professionals involved in the introduction of an innovative IP/D and patients who are eligible to undergo it. The clinician responsible for its introduction will initially be identified, with additional healthcare professionals being identified by the snowball technique, in which interviewees provide the names of other contacts. Patients will be eligible to take part in the study if they are being offered or have recently undergone (within three months from discharge) an innovative IP/D. Patients will be identified by the surgical team and asked to consider taking part in the study. Only patients who are over 18 years old and have the capacity to consent will be eligible. Patients and healthcare professionals will be asked to provide written consent to taking part in the individual components of the study (e.g. audio recording of consultation, interviews, etc).

Data collection

Each case study involved the collection of multiple data sources: semi-structured interviews with healthcare professionals and patients, audio recording consultations between healthcare professionals and patients, video-recording and observing the procedure, and capturing clinical and patient information (these are described below). Data collection and analysis will proceed in parallel, with emerging findings informing data collection.

Semi-structured interviews

Separate topic guides will be developed for the different types of interviews (see Table 1). Discussions will be guided by topic guides to ensure that the discussions cover the same core issues[27 28], whilst allowing for probing questions for each participant to enable new issues of importance to be discussed further [29 30]. Topic guides will be adapted as analysis progresses[27 31], to enable exploration of insights identified across and within case studies. Reflective notes will also be made during the interviews, taking account of the interviewers' thoughts and ideas. All interviews will be audio-recorded using an encrypted recorder.

Table 1: Interviews within each case study

For peer review only - http://bmjopen.bmj.com/site/about/guidelines.xhtml

Interview type	Purpose of the interview
**'Background' interviews with healthcare professionals**	A lead clinician responsible for the introduction of the innovative IP/D will take part in an initial, scene-setting interview to understand what the procedure involves, how it is innovative, any evidence for supporting the use of the procedure, and views as to what patients should be told. Additional healthcare professionals (e.g. surgeons, nurses, anaesthetists) may also be interviewed to explore any aligning or contrasting experiences and views of the procedure.
**Post-operative interviews with surgical teams**	Surgical teams, including the clinical lead, will be asked to take part in interviews throughout the study to investigate how the IP/D is refined over time, deviations from the planned surgery and plans for modifying the procedure. Where possible, the interview will be conducted immediately after the procedure.
**Patient interview**	Interviews with patients will explore views on the presentation of information provided about the procedure during consultations, reasons underlying decisions to accept or decline the procedure, views/understanding of innovation and (if relevant) their experience of undergoing the procedure and subsequent recovery.
**End of case study interview**	Lead clinicians and other healthcare professionals will be invited to take part in a final interview at the end of the case study period or at the point they, and the study team, consider the procedure to have stabilised (i.e. no longer undergoing significant modifications). Interviews will explore their views of, and future plans for, the procedure (i.e. further dissemination, training and evaluation).

For peer review only

Audio recording consultations between healthcare professionals and patients

Qualitative analyses of audio recorded consultations have been successfully applied to the study of informed consent to clinical trial participation [32 33] and will be used in this study to explore how information about innovative IP/Ds is communicated and received. Consultations during which procedures of interest are discussed with eligible patients (including telephone or video-link conversations) will be audio-recorded using an encrypted audio recorder.

Video-recording and observing the procedure

Consecutive invasive procedures for each case study will be video recorded to investigate the evolution of modification(s) in each procedure over time, and explore how to recognise when a procedure has sufficiently stabilised. Video recording has been done successfully in several other surgical studies [34 35]. Video recordings will capture the entire procedure so that the research team can deconstruct and identify the key components of the procedure, investigate how the procedure is refined and identify how unexpected events, and patient or contextual factors are dealt with. The field-of-view will capture only the area of interest/surgical site and not identify any healthcare professionals or patients. Concurrent observation will be non-participant in nature and involve the compilation of field notes relating to verbal and non-verbal communication and contextual factors.

Clinical and patient data collection

Data about the patients, procedure and nature of the modifications, and clinical outcomes will be recorded from the above data sources and supplemented by hospital records.

Data analysis

In line with the study objectives, a summary of which data sources will be included in the different analyses is in Table 2.

Table 2: How the different data sources will be used

**Aim:**	**To explore how innovative invasive procedures and/or devices are introduced into NHS hospitals**		
**Specific objectives:**	To understand how information is communicated to and understood by patients	To understand how procedures evolve over time	To understand how outcomes are selected, measured, and reported
	↓	↓	↓
**Data sources included in analysis:**	'Background' interviews<br>Audio recording consultations<br>Patient interviews	'Background' interviews<br>Video-recording procedures<br>Non-participant observations<br>Post-operative interviews<br>Clinical and patient information<br>End of case study interview	'Background' interviews<br>Video-recording procedures<br>Non-participant observations<br>Post-operative interviews<br>Clinical and patient information<br>End of case study interview

Data analysis of interviews

All audio data will be transcribed verbatim in full or in a targeted manner (where only relevant sections of the consultation will be transcribed). Transcripts will de-identified, checked against original recordings, and imported into software (QSR International, USA). Interview data will be systematically assigned codes and analysed using constant comparison methods derived from grounded theory methodology[22 36].

Data analysis of consultations

We will draw from content, thematic and targeted conversation analytic approaches to investigate the delivery of information during the consultation appointments[22 37-39]. Analysis will centre on exploring patient-healthcare professional interactions (e.g. analysis of patient requests for clarification), the type of information communicated to patients (e.g. potential risks/benefits, uncertainties), patients' responses to being offered the procedure and their involvement in the decision-making process. Comparisons will be made between the interview data (what surgeons and patients report of the consultation) and the consultation audio data (what transpires in the consultation) across different data sources.

Data analysis of video recordings and non-participant observations

Videos will time be analysed in chronological order to understand how the procedure evolves over time. The videos of surgical procedures will be viewed unedited and transcribed from beginning to end. This will involve the researcher watching and re-watching the recording to familiarise

For peer review only - http://bmjopen.bmj.com/site/about/guidelines.xhtml

themselves with the procedure and to document movements, instruments and actions that were captured on the screen[34]. Movements and actions from the video transcript will be grouped together into operative steps, which will then generate a step-by-step overview of the procedure. Notes from non-participant observation, and issues discussed in the health professional interviews, will be added to the respective operative step(s) from the video recording.

Ethics and dissemination

The study has been reviewed and approved (HRA and Health and Care Research Wales) by the Frenchay Research Ethics Committee (Ref 18/SW/0277). Study results will be presented to different audiences including academic, clinical, and lay members of the public. It is hoped that this work will facilitate a better understanding of the process of surgical innovation, so that new methods for efficient, safe and timely design and conduct of innovative invasive IP/Ds can be developed. Specifically, it is hoped that this will be achieved by: i) optimising patient information provision and informed consent for innovative IP/Ds; ii) improving the evaluation of surgical interventions and being able to establish studies and study designs that precede and include surgical RCTs; iii) establishing guidance for the selection and reporting of outcomes of innovative IP/Ds; and iv) developing a reporting and sharing platform for innovative procedures.

Patient and public involvement

The current study comprises a core component of the work undertaken within the National Institute for Health Research (NIHR) Bristol Biomedical Research Centre (BRC) Surgical Innovation theme, which aims to improve the safe and transparent translation of innovative IP/Ds to clinical practice. A patient and public involvement (PPI) group will be established, in which patients who have undergone surgery are asked about their views regarding how new surgical procedures are undertaken in NHS clinical practice. The PPI group will have involvement in the Lotus study from inception to its completion. This will include providing feedback on study aims, data collection plans and study documentation as well as contributing to the data analysis and dissemination of findings.

Author contributions

DE, NB and JMB developed the idea for the current study. DE drafted the first version of the protocol, with critical revision from JMB. All authors commented on versions of the protocol and gave approval for the manuscript to be submitted. The NIHR Bristol Biomedical Research Centre Surgical Innovation theme is directed by JMB (theme lead).

For peer review only - http://bmjopen.bmj.com/site/about/guidelines.xhtml

Disclosure: The authors declare no conflict of interest.

For peer review only

For peer review only - http://bmjopen.bmj.com/site/about/guidelines.xhtml

References

1. Abbott T, Fowler A, Dobbs T, et al. Frequency of surgical treatment and related hospital procedures in the UK: a national ecological study using hospital episode statistics. 2017;119(2):249-57.
2. Biffl WL, Spain DA, Reitsma AM, et al. Responsible Development and Application of Surgical Innovations: A Position Statement of the Society of University Surgeons. *J Am Coll Surg* 2008;206(6):1204-09. doi: https://doi.org/10.1016/j.jamcollsurg.2008.02.011
3. Birchley G, Huxtable R, Ives J, et al. Have We Made Progress in Identifying (Surgical) Innovation? *The American Journal of Bioethics* 2019;19(6):25-27. doi: 10.1080/15265161.2019.1602181
4. Hutchison K, Rogers W, Eyers A, et al. Getting Clearer about surgical innovation: a new definition and a new tool to support responsible practice. *Ann Surg* 2015;262(6):949-54.
5. Angelos P. Ethics and surgical innovation: challenges to the professionalism of surgeons. *International Journal of Surgery*;11:S2-S5. doi: 10.1016/S1743-9191(13)60003-5
6. Lotz M. Surgical innovation as sui generis surgical research. *Theor Med Bioeth* 2013;34(6):447-59.
7. Kerr RJTBotRCoSoE. The future of surgery. 2019;101(7):264-67.
8. Reitsma A, Moreno J. *J Am Coll Surg* 2002;194(null):792.
9. Reitsma AM, Moreno JD. *The American Journal of Bioethics* 2003;3(null):49.
10. Broekman ML, Carriere ME, Bredenoord AL. Surgical innovation: the ethical agenda: A systematic review. *Medicine (Baltimore)* 2016;95(25):e3790. doi: 10.1097/md.0000000000003790 [published Online First: 2016/06/24]
11. Barkun JS, Aronson JK, Feldman LS, et al. *Lancet* 2009;374(null):1089.
12. Khachane A, Philippou Y, Hirst A, et al. Appraising the uptake and use of the IDEAL Framework and Recommendations: A review of the literature. *Int J Surg* 2018;57:84-90. doi: 10.1016/j.ijsu.2018.07.008 [published Online First: 2018/08/12]
13. Macefield R, Wilson N, Hoffmann C, et al. Outcome selection, measurement and reporting for new surgical procedures and devices: a systematic review of IDEAL/IDEAL-D studies to inform development of a core outcome set. 2020;4(6):1072-83.
14. Bilbro NJBJoS. New reporting guidelines for IDEAL studies. 2020;107(10):1241-42.
15. Cousins S, Richards H, Zahra J, et al. Introduction and adoption of innovative invasive procedures and devices in the NHS: an in-depth analysis of written policies and qualitative interviews (the INTRODUCE study protocol). 2019;9(8):e029963. doi: 10.1136/bmjopen-2019-029963 %J BMJ Open
16. Dyer C. Robot assisted surgery is blamed for heart patient's death: British Medical Journal Publishing Group, 2018.
17. Zahra J, Paramasivan S, Blencowe NS, et al. Discussing surgical innovation with patients: a qualitative study of surgeons' and governance representatives' views. 2020;10(11):e035251.
18. Cumberlege JJUGR, https://www.immdsreview. org. uk/Report. html. First Do No Harm: The Report of the Independent Medicines and Medical Devices Safety Review. 2020
19. Di Paolo M, Boggi U, Turillazzi EJTBjos. Bioethical approach to robot-assisted surgery. 2019;106(10):1271-72.
20. Robson C, McCartan K. Real world research: John Wiley & Sons 2016.
21. Yin RKJTShoasrm. How to do better case studies. 2009;2:254-82.
22. Glaser BG, Strauss AL. The Discovery of Grounded Theory. Aldine Transaction: USA 1967.
23. Krippendorff KJHcr. Reliability in content analysis: Some common misconceptions and recommendations. 2004;30(3):411-33.
24. Miles MB, Huberman AM, Huberman MA, et al. Qualitative data analysis: An expanded sourcebook: sage 1994.
25. Cousins S, Blencowe NS, Blazeby JMJBo. What is an invasive procedure? A definition to inform study design, evidence synthesis and research tracking. 2019;9(7):e028576.

For peer review only

26. Medical Devices Regulations 2002 (SI 2002 No 618 aaUM. [Available from: https://www.gov.uk/guidance/medical-devices-how-to-comply-with-the-legal-requirements accessed 11th January 2021. .
27. Smith JAJRmip. Semi-structured interviewing and qualitative analysis. 1995
28. Britten NJB. Qualitative research: qualitative interviews in medical research. 1995;311(6999):251-53.
29. Rubin HJ, Rubin IS. Qualitative interviewing: The art of hearing data: sage 2011.
30. Hammersley P, Atkinson M. Ethnography: principles and practice. London: Routledge 1983.
31. Charmaz K, Belgrave LJTShoirTcotc. Qualitative interviewing and grounded theory analysis. 2012;2:347-65.
32. Donovan JL, Rooshenas L, Jepson M, et al. Optimising recruitment and informed consent in randomised controlled trials: the development and implementation of the Quintet Recruitment Intervention (QRI). *Trials* 2016;17(1):283. doi: 10.1186/s13063-016-1391-4
33. Elliott D, Husbands S, Hamdy FC, et al. Understanding and Improving Recruitment to Randomised Controlled Trials: Qualitative Research Approaches. *Eur Urol* doi: 10.1016/j.eururo.2017.04.036
34. Blencowe NS, Blazeby JM, Donovan JL, et al. Novel ways to explore surgical interventions in randomised controlled trials: applying case study methodology in the operating theatre. 2015;16(1):589.
35. Blencowe NS, Skilton A, Gaunt D, et al. Protocol for developing quality assurance measures to use in surgical trials: an example from the ROMIO study. 2019;9(3):e026209.
36. Braun V, Clarke V. Using thematic analysis in psychology. *Qualitative Research in Psychology* 2006;3(2):77-101. doi: 10.1191/1478088706qp063oa
37. Seale C, Silverman DJTEJoPH. Ensuring rigour in qualitative research. 1997;7(4):379-84.
38. Jepson M, Elliott D, Conefrey C, et al. An observational study showed that explaining randomization using gambling-related metaphors and computer-agency descriptions impeded randomized clinical trial recruitment. 2018;99:75-83.
39. Kalekin-Fishman D. Review: David Silverman (2001). Interpreting Qualitative Data: Methods for Analysing Talk, Text and Interaction. *2001* 2001;2(3) doi: 10.17169/fqs-2.3.923 %J Forum Qualitative Sozialforschung / Forum: Qualitative Social Research [published Online First: 2001-09-30]

For peer review only - http://bmjopen.bmj.com/site/about/guidelines.xhtml

BMJ Open

Using qualitative research methods to understand how surgical procedures and devices are introduced into NHS hospitals: The Lotus study protocol

Journal:	*BMJ Open*
Manuscript ID	bmjopen-2021-049234.R1
Article Type:	Protocol
Date Submitted by the Author:	22-Jun-2021
Complete List of Authors:	Elliott, Daisy; University of Bristol<br>Blencowe, Natalie; University of Bristol, Centre for Surgical Research<br>Cousins, Sian; University of Bristol, Population Health Sciences<br>Zahra, Jesmond; University of Bristol, Centre for Surgical Research<br>Skilton, Anni; University of Bristol<br>Mathews, Johnny; University of Bristol, Population Health Sciences<br>Paramasivan, Sangeetha ; University of Bristol<br>Hoffmann, Christin; University of Bristol<br>McNair, Angus; University of Bristol, Centre for Surgical Research, Bristol Medical School: Population Health Sciences; North Bristol NHS Trust, GI Surgery<br>Ochieng, Cynthia; University of Bristol<br>Richards, Hollie; University of Bristol, Population Health Sciences<br>Hossaini, Sina; University of Bristol<br>Scroggie, Darren; University of Bristol<br>Main, Barry; University of Bristol, School of Social and Community Medicine<br>Potter, Shelley; University of Bristol<br>Avery, Kerry; University of Bristol<br>Donovan, Jenny; University of Bristol<br>Blazeby, Jane; University of Bristol
**Primary Subject Heading**:	Health services research
Secondary Subject Heading:	Qualitative research
Keywords:	SURGERY, QUALITATIVE RESEARCH, Quality in health care < HEALTH SERVICES ADMINISTRATION & MANAGEMENT

SCHOLARONE™
Manuscripts

I, the Submitting Author has the right to grant and does grant on behalf of all authors of the Work (as defined in the below author licence), an exclusive licence and/or a non-exclusive licence for contributions from authors who are: i) UK Crown employees; ii) where BMJ has agreed a CC-BY licence shall apply, and/or iii) in accordance with the terms applicable for US Federal Government officers or employees acting as part of their official duties; on a worldwide, perpetual, irrevocable, royalty-free basis to BMJ Publishing Group Ltd ("BMJ") its licensees and where the relevant Journal is co-owned by BMJ to the co-owners of the Journal, to publish the Work in this journal and any other BMJ products and to exploit all rights, as set out in our licence.

The Submitting Author accepts and understands that any supply made under these terms is made by BMJ to the Submitting Author unless you are acting as an employee on behalf of your employer or a postgraduate student of an affiliated institution which is paying any applicable article publishing charge ("APC") for Open Access articles. Where the Submitting Author wishes to make the Work available on an Open Access basis (and intends to pay the relevant APC), the terms of reuse of such Open Access shall be governed by a Creative Commons licence – details of these licences and which Creative Commons licence will apply to this Work are set out in our licence referred to above.

Other than as permitted in any relevant BMJ Author's Self Archiving Policies, I confirm this Work has not been accepted for publication elsewhere, is not being considered for publication elsewhere and does not duplicate material already published. I confirm all authors consent to publication of this Work and authorise the granting of this licence.

For peer review only

Using qualitative research methods to understand how surgical procedures and devices are introduced into NHS hospitals: The Lotus study protocol

Submission category: Protocol

Authors: Elliott D[1,], Blencowe NS[1, 2], Cousins S[1], Zahra J[1], Skilton A[1], Mathews JA[1], Paramasivan S[1], Hoffmann C[1], McNair AGK[1,3], Ochieng C[1], Richards H[1], Hossaini S[1], Scroggie D[1], Main BG[1,2], Potter S[1], Avery KNL[1], Donovan JL [4], Blazeby JM[1, 2]

Affiliations:

[1]National Institute for Health Research Bristol Biomedical Research Centre, Surgical Innovation Theme, Centre for Surgical Research, University of Bristol, 39 Whatley Road, Clifton, Bristol, UK, BS8 2PS

[2] University Hospitals Bristol and Weston NHS Foundation Trust, Bristol, UK, BS2 8HW

[3] North Bristol NHS Trust, Bristol, UK, BS10 5NB

[4] Population Health Sciences, Bristol Medical School, University of Bristol, 39 Whatley Road, Clifton, Bristol, UK, BS8 2PS

Correspondence to: Daisy Elliott [daisy.elliott@bristol.ac.uk]

Running title: LOTUS STUDY PROTOCOL

Keywords: Surgery; Innovation; Qualitative

Funding statement: This study was supported by the NIHR Biomedical Research Centre at University Hospitals Bristol and Weston NHS Foundation Trust and the University of Bristol (BRC-1215-20011). The views expressed in this publication are those of the author(s) and not necessarily those of the NHS, the National Institute for Health Research or the Department of Health and Social Care. JLD and JMB are NIHR Senior Investigators. NSB and AGKM are MRC Clinician Scientists.

Word count: 1908 (excluding title page, abstract, tables and references)

For peer review only

Abstract

Introduction:

The development of innovative invasive procedures and devices are essential to improving outcomes in healthcare. However, how these are introduced into practice has not been studied in detail. The Lotus study will follow a wide range of 'case studies' of new procedures and/or devices being introduced into NHS trusts to explore what information is communicated to patients, how procedures are modified over time, and how outcomes are selected and reported.

Methods and analysis:

This qualitative study will use ethnographic approaches to investigate how new invasive procedures and/or devices are introduced. Consultations in which the innovation is discussed will be audio-recorded to understand information provision practice. To understand if and how procedures evolve, they will be video recorded and non-participant observations will be conducted. Post-operative interviews will be conducted with the innovating team and patients who are eligible for the intervention. Audio recordings will be audio-recorded, transcribed verbatim and analysed thematically using constant comparison techniques. Video recordings will be reviewed to deconstruct procedures into key components and document how the procedure evolves. Comparisons will be made between the different data sources.

Ethics and dissemination:

The study protocol has HRA and Health and Care Research Wales approval (Ref 18/SW/0277). Results will be disseminated at appropriate conferences and will be published in peer-reviewed journals. The findings of this study will provide a better understanding of how innovative invasive procedures and/or devices are introduced into practice.

(233 words)

For peer review only

Strengths and limitations of this study

- Studying innovation in real-time is novel and enables in-depth and contemporaneous insights
- Including a range of specialities and hospitals will provide a broad overview of how innovative invasive procedures and devices are introduced into NHS hospitals
- Triangulation of multiple methods of data collection will facilitate a comprehensive understanding of surgical innovation and help to validate findings

For peer review only - http://bmjopen.bmj.com/site/about/guidelines.xhtml

- Findings may be limited by snowball sampling and the self-selecting healthcare professionals who agree to take part in the study
- Although this study will provide important and rich insights into practices in the United Kingdom (UK), findings may not be generalisable to other countries and different healthcare systems

For peer review only

For peer review only - http://bmjopen.bmj.com/site/about/guidelines.xhtml

Introduction

At least 12.5 million invasive procedures are delivered in the UK annually[1]. Innovation is critical and constantly occurring because of the drive to improve a range of outcomes. Common targets include using less invasive techniques; shortening hospital stays; improving patient outcomes; or creating novel therapeutic options[2-4]. Innovation also frequently occurs because of a healthcare professional's desire to pioneer new techniques and to be recognised as a leader in the field [4]. An independent review by the Royal College of Surgeons of England has predicted that surgery will undergo a massive transformation because of developments in new technologies such as robotics, artificial intelligence, genomics and digital technologies, and concluded that the world of surgery is embarking on a time of innovation[5].

Unlike the pharmaceutical industry, where there are extensive regulatory and ethical requirements[6-8], the introduction of innovative invasive procedures and/or devices (IP/Ds) is not adequately regulated. The IDEAL framework [9] outlines five consecutive stages of development, evaluation and dissemination of innovative IP/Ds, although its uptake has been slow [10 11] and it is often incorrectly followed [12]. Furthermore, whilst innovative IP/Ds should be introduced in the context of formal research studies or in accordance with local hospital policies and processes[13], ethical approval is rarely gained [8] and procedures can be introduced without any formal governance from local hospitals [14 15]. Important questions have also been raised surrounding informed consent for innovative IP/Ds. For instance, an inquest into the death of the first patient in the UK to undergo robotically assisted heart surgery found the patient had not been fully informed about the comparative risks of robotic versus conventional open surgery[14].

A recent independent review has highlighted the need for better governance and evaluation of surgical innovation [16]. As little is known about mechanisms of how new IP/Ds are introduced into practice[17], a greater understanding of how innovative procedures are introduced is imperative to developing guidance and regulation. Complex phenomena such as innovative IP/Ds can be explored in-depth using qualitative methodology so that rich insights and experiences of innovation from a number of perspectives can be captured and explored in detail within their original setting[18 19]. The Lotus study will use qualitative research methods to provide in-depth, real-time insights to how innovative IP/Ds are introduced into NHS hospitals. Specific objectives of the Lotus study are to explore: i) how information is communicated to patients and how patients understand this information in order to inform their decision-making process; ii) how procedures evolve over time; and iii) how outcomes are selected, measured and reported.

For peer review only - http://bmjopen.bmj.com/site/about/guidelines.xhtml

For peer review only

Methods and analysis

Design

The Lotus study will use ethnographic approaches to investigate how new invasive IP/Ds are introduced in the NHS. A grounded theory methodology will enable the inductive identification of themes that are derived or grounded in the data [20 21]. Its central principle is of constant comparison, where new findings are systematically compared with existing data so that similarities and differences can be identified through the ongoing assimilation of data [20 22].

Case study eligibility

A senior academic surgeon and the BRC Surgical Innovation theme lead (JMB) will approach individuals from NHS trusts, NHS New Procedure Committees and funding bodies to identify innovative IP/Ds. Firstly, an in-depth qualitative interview with the innovator (a 'background' interview) will be conducted to learn more about the innovative IP/D. Alongside this, the research team will undertake a literature review to identify existing published articles on the procedure. A data extraction form will be developed specifically for the review to record details of each article (relating to authors, year, country of origin, study design, participants, and number of participating centres).

Procedures will be eligible if they are deemed to be innovative IP/Ds (see Table 1). The background interview and review of published evidence will be examined together by the study team and reasons for exclusion will be documented (i.e. if published evidence was such that the outcomes could reasonably be considered to have been systematically evaluated and reported[23], or that the number of procedures already delivered in the organisation had surpassed that where it would have been viable to collect data about its introduction).

Table 1: Case study eligibility

Procedures will be eligible for the study if they are deemed to be:	
**Innovative**	• A new or modified procedure that differs from currently accepted local practice, the outcomes of which have not been fully systematically evaluated and reported in a standardised manner, and which may entail unknown risks to the patient[23 24]. This can include the introduction of entirely new procedures as well as undertaking modifications to existing techniques.
**And:**	

For peer review only - http://bmjopen.bmj.com/site/about/guidelines.xhtml

**An invasive procedure or a device**	• An invasive procedure will be defined as one in which purposeful and deliberate access to the body is gained via an incision or percutaneous puncture, instrumentation is used in addition to a puncture needle, or instrumentation occurs via a natural orifice. It begins when entry to the body is gained and ends when the instrument is removed and/or the skin is closed. It is performed by trained healthcare professionals using instruments, which include, but are not limited to, endoscopes, catheters, scalpels, scissors, devices and tubes[25].<br>• A device is defined as any instrument, apparatus, appliance, software, material or other article, whether used alone or in combination, which is intended by the manufacturer to be used for human beings[26].

We will aim to follow a range of case studies to capture innovation in different contexts, including varying stages of innovation (as identified by the IDEAL framework[9]), type of innovation (procedure or device), surgical specialty, and NHS trust type (e.g. geographical area, foundation status and acute trust type). Where possible, we will try to identify innovative IP/Ds that are due to be introduced into practice to enable us to capture data from the first time they are performed.

Whilst the exact numbers of participants recruited will vary depending on the frequency of procedures and the number of healthcare professionals involved, we estimate that for each case study we will recruit a minimum of five patients and three healthcare professionals. Although we anticipate approximately 5-10 complete case studies will be included, identification of new case studies will ultimately continue until additional data are not adding anything new to the analytical framework and theoretical saturation is felt to have been achieved. In addition, specific study components may be carried out as stand-alone elements (e.g. video-recordings of operations without the interviews and audio-recordings of consultations, or vice versa) for some procedures.

Recruitment and sampling

For each case study, participants will include healthcare professionals involved in the introduction of an innovative IP/D and patients who are eligible to undergo it. The clinician responsible for its introduction will initially be identified, with additional healthcare professionals being identified by the snowball technique, in which interviewees provide the names of other contacts. Patients will be eligible to take part in the study if they are being offered or have recently undergone (within three months from discharge) an innovative IP/D. Patients will be identified by the surgical team and asked

For peer review only - http://bmjopen.bmj.com/site/about/guidelines.xhtml

to consider taking part in the study. Only patients who are over 18 years old and have the capacity to consent will be eligible. Patients and healthcare professionals will be asked to provide written consent to taking part in the individual components of the study (e.g. audio recording of consultation, interviews, etc).

Data collection

Each case study involved the collection of multiple data sources: semi-structured interviews with healthcare professionals and patients, audio recording consultations between healthcare professionals and patients, video-recording and observing the procedure, and capturing clinical and patient information (these are described below). Data collection and analysis will proceed in parallel, with emerging findings informing data collection.

Semi-structured interviews

Separate topic guides will be developed for the different types of interviews (see Table 2). Discussions will be guided by topic guides to ensure that the discussions cover the same core issues[27 28], whilst allowing for probing questions for each participant to enable new issues of importance to be discussed further [29 30]. Topic guides will be adapted as analysis progresses[27 31], to enable exploration of insights identified across and within case studies. Reflective notes will also be made during the interviews, taking account of the interviewers' thoughts and ideas. All interviews will be audio-recorded using an encrypted recorder.

Table 2: Interviews within each case study

Interview type	Purpose of the interview
**'Background' interviews with healthcare professionals**	A lead clinician responsible for the introduction of the innovative IP/D will take part in an initial, scene-setting interview to understand what the procedure involves, how it is innovative, any evidence for supporting the use of the procedure, and views as to what patients should be told. Additional healthcare professionals (e.g. surgeons, nurses, anaesthetists and representatives involved in regulating the introduction of innovative IP/D at trust or national levels) may also be interviewed to explore any aligning or contrasting experiences and views of the procedure.
**Post-operative interviews with surgical teams**	Surgical teams, including the clinical lead, will be asked to take part in interviews throughout the study to investigate how the IP/D is refined over time, deviations from the planned surgery and plans for modifying the procedure. Where possible, the interview will be conducted immediately after the procedure.
**Patient interview**	Interviews with patients will explore views on the presentation of information provided about the procedure during consultations, reasons underlying decisions to accept or decline the procedure, views/understanding of innovation and (if relevant) their experience of undergoing the procedure and subsequent recovery.
**End of case study interview**	Lead clinicians and other healthcare professionals will be invited to take part in a final interview at the end of the case study period or at the point they, and the study team, consider the procedure to have stabilised (i.e. no longer undergoing significant modifications). Interviews will explore their views of, and future plans for, the procedure (i.e. further dissemination, training and evaluation).

For peer review only - http://bmjopen.bmj.com/site/about/guidelines.xhtml

Audio recording consultations between healthcare professionals and patients

Qualitative analyses of audio recorded consultations have been successfully applied to the study of informed consent to clinical trial participation [32 33] and will be used in this study to explore how information about innovative IP/Ds is communicated and received. Consultations during which procedures of interest are discussed with eligible patients (including telephone or video-link conversations) will be audio-recorded using an encrypted audio recorder.

Video-recording and observing the procedure

Consecutive invasive procedures for each case study will be video recorded to investigate the evolution of modification(s) in each procedure over time, and explore how to recognise when a procedure has sufficiently stabilised. Video recording has been done successfully in several other surgical studies [34 35] and has been found to be feasible and acceptable to the surgeon innovators. It is intended that video recordings will capture the entire procedure. Recordings taken from laparoscopic or robotic video feeds will comprise only of unidentifiable intra-operative footage. In the case of 'open' surgery (non-minimally invasive), the field-of-view will capture only the area of interest/surgical site and not identify any healthcare professionals or patients. Audio will not be captured. Should any patient or staff identifiers be inadvertently captured, these will be removed in post-production by the study research photographer. Concurrent observation (where possible) will be non-participant in nature and involve the compilation of field notes relating to verbal and non-verbal communication and contextual factors.

Clinical and patient data collection

Data about the patients, procedure and nature of the modifications, and clinical outcomes will be recorded from the above data sources and supplemented by hospital records.

Data analysis

In line with the study objectives, a summary of which data sources will be included in the different analyses is in Table 3.

Table 3: How the different data sources will be used

**Aim:**	**To explore how innovative invasive procedures and/or devices are introduced into NHS hospitals**		
**Specific objectives:**	To understand how information is communicated to and understood by patients	To understand how procedures evolve over time	To understand how outcomes are selected, measured, and reported
	↓	↓	↓

For peer review only

**Data sources included in analysis:**	'Background' interviews<br>Audio recording consultations<br>Patient interviews	'Background' interviews<br>Video-recording procedures<br>Non-participant observations<br>Post-operative interviews<br>Clinical and patient information<br>End of case study interview	'Background' interviews<br>Video-recording procedures<br>Non-participant observations<br>Post-operative interviews<br>Clinical and patient information<br>End of case study interview

Data analysis of interviews

All audio data will be transcribed verbatim in full or in a targeted manner (where only relevant sections of the consultation will be transcribed). Transcripts will de-identified, checked against original recordings, and imported into software (QSR International, USA). Interview data will be systematically assigned codes and analysed using constant comparison methods derived from grounded theory methodology[20 36].

Data analysis of consultations

We will draw from content, thematic and targeted conversation analytic approaches to investigate the delivery of information during the consultation appointments[20 37-39]. Analysis will centre on exploring patient-healthcare professional interactions (e.g. analysis of patient requests for clarification), the type of information communicated to patients (e.g. potential risks/benefits, uncertainties), patients' responses to being offered the procedure and their involvement in the decision-making process. Comparisons will be made between the interview data (what surgeons and patients report of the consultation) and the consultation audio data (what transpires in the consultation) across different data sources.

Data analysis of video recordings and non-participant observations

Each video will be viewed, from beginning to end, by an academic surgeon. This will involve watching and re-watching the recording to familiarise themselves with the procedure and to document movements, instruments, use of any assistants and actions that were captured on the screen[34]. The video will be analysed by the procedural components. This will be done with reference to findings from healthcare professional interviews, alongside an understanding of the existing publications and surgical knowledge of the procedure. Essentially, the research team will 'deconstruct' the intervention into its component parts[34 40 41]. Where available, notes from non-participant observations will be added to the respective operative step(s) from the video recording. Other intra-operative data may be viewed in conjunction during video analysis, such as length of procedure, port-placement time, time on operative console and blood loss. In the case of innovative robotic procedures, images of port-site placements will also be captured and reviewed. This will enable the research team to document how the component parts were delivered, as well as components which

For peer review only - http://bmjopen.bmj.com/site/about/guidelines.xhtml

those were not delivered or modified compared to what was anticipated. It will also identify how unexpected events are managed intra-operatively, and how many patient (anatomical) or contextual (theatre) factors are dealt with. Videos will be analysed in chronological order to understand how the procedure evolves over time. Taken together, this will allow the research team to investigate how the procedure is refined and changed. Where changes to the procedure are made, post - operative interviews with the surgeons will be conducted to explore the rationale for the modification. This will compile a descriptive analysis of the novel procedure as a case study.

Ethics and dissemination

The study has been reviewed and approved (HRA and Health and Care Research Wales) by the Frenchay Research Ethics Committee (Ref 18/SW/0277) on 31/12/2018. The initial study length will be five years. Study results will be presented to different audiences including academic, clinical, and lay members of the public. It is hoped that this work will facilitate a better understanding of the process of surgical innovation, so that new methods for efficient, safe and timely design and conduct of innovative invasive IP/Ds can be developed. Specifically, it is hoped that this will be achieved by: i) optimising patient information provision and informed consent for innovative IP/Ds; ii) improving the evaluation of surgical interventions and being able to establish studies and study designs that precede and include surgical RCTs; iii) establishing guidance for the selection and reporting of outcomes of innovative IP/Ds; and iv) developing a reporting and sharing platform for innovative procedures (an 'e-platform').

Patient and public involvement

The current study comprises a core component of the work undertaken within the National Institute for Health Research (NIHR) Bristol Biomedical Research Centre (BRC) Surgical Innovation theme, which aims to improve the safe and transparent translation of innovative IP/Ds to clinical practice. A patient and public involvement (PPI) group will be established, in which patients who have undergone surgery are asked about their views regarding how new surgical procedures are undertaken in NHS clinical practice. The PPI group will have involvement in the Lotus study from inception to its completion. This will include providing feedback on study aims, data collection plans and study documentation as well as contributing to the data analysis and dissemination of findings.

Author contributions

DE, NB and JMB developed the idea for the current study. DE drafted the first version of the protocol, with critical revision from JMB. NSB, SC, JZ, AS, JM, SP, CH, AM, CO, HR, SH, DS, BGM, SP,

For peer review only - http://bmjopen.bmj.com/site/about/guidelines.xhtml

KNLA and JLD commented on versions of the protocol and gave approval for the manuscript to be submitted. The NIHR Bristol Biomedical Research Centre Surgical Innovation theme is directed by JMB (theme lead).

Disclosure: The authors declare no conflict of interest.

For peer review only

For peer review only - http://bmjopen.bmj.com/site/about/guidelines.xhtml

References

1. Abbott T, Fowler A, Dobbs T, et al. Frequency of surgical treatment and related hospital procedures in the UK: a national ecological study using hospital episode statistics. 2017;119(2):249-57.
2. Angelos P. Ethics and surgical innovation: challenges to the professionalism of surgeons. *International Journal of Surgery*;11:S2-S5. doi: 10.1016/S1743-9191(13)60003-5
3. Lotz M. Surgical innovation as sui generis surgical research. *Theor Med Bioeth* 2013;34(6):447-59.
4. Hutchison K, Rogers W, Eyers A, et al. Getting Clearer about surgical innovation: a new definition and a new tool to support responsible practice. *Ann Surg* 2015;262(6):949-54.
5. Kerr RJTBotRCoSoE. The future of surgery. 2019;101(7):264-67.
6. Reitsma A, Moreno J. *J Am Coll Surg* 2002;194(null):792.
7. Reitsma AM, Moreno JD. *The American Journal of Bioethics* 2003;3(null):49.
8. Broekman ML, Carriere ME, Bredenoord AL. Surgical innovation: the ethical agenda: A systematic review. *Medicine (Baltimore)* 2016;95(25):e3790. doi: 10.1097/md.0000000000003790 [published Online First: 2016/06/24]
9. Barkun JS, Aronson JK, Feldman LS, et al. *Lancet* 2009;374(null):1089.
10. Khachane A, Philippou Y, Hirst A, et al. Appraising the uptake and use of the IDEAL Framework and Recommendations: A review of the literature. *Int J Surg* 2018;57:84-90. doi: 10.1016/j.ijsu.2018.07.008 [published Online First: 2018/08/12]
11. Macefield R, Wilson N, Hoffmann C, et al. Outcome selection, measurement and reporting for new surgical procedures and devices: a systematic review of IDEAL/IDEAL-D studies to inform development of a core outcome set. 2020;4(6):1072-83.
12. Bilbro NJBJoS. New reporting guidelines for IDEAL studies. 2020;107(10):1241-42.
13. Cousins S, Richards H, Zahra J, et al. Introduction and adoption of innovative invasive procedures and devices in the NHS: an in-depth analysis of written policies and qualitative interviews (the INTRODUCE study protocol). 2019;9(8):e029963. doi: 10.1136/bmjopen-2019-029963 %J BMJ Open
14. Dyer C. Robot assisted surgery is blamed for heart patient’s death: British Medical Journal Publishing Group, 2018.
15. Zahra J, Paramasivan S, Blencowe NS, et al. Discussing surgical innovation with patients: a qualitative study of surgeons’ and governance representatives’ views. 2020;10(11):e035251.
16. Cumberlege JJUGR, https://www. immdsreview. org. uk/Report. html. First Do No Harm: The Report of the Independent Medicines and Medical Devices Safety Review. 2020
17. Di Paolo M, Boggi U, Turillazzi EJTBjos. Bioethical approach to robot-assisted surgery. 2019;106(10):1271-72.
18. Robson C, McCartan K. Real world research: John Wiley & Sons 2016.
19. Yin RKJTShoasrm. How to do better case studies. 2009;2:254-82.
20. Glaser BG, Strauss AL. The Discovery of Grounded Theory. Aldine Transaction: USA 1967.
21. Krippendorff KJHcr. Reliability in content analysis: Some common misconceptions and recommendations. 2004;30(3):411-33.
22. Miles MB, Huberman AM, Huberman MA, et al. Qualitative data analysis: An expanded sourcebook: sage 1994.
23. Biffl WL, Spain DA, Reitsma AM, et al. Responsible Development and Application of Surgical Innovations: A Position Statement of the Society of University Surgeons. *J Am Coll Surg* 2008;206(6):1204-09. doi: https://doi.org/10.1016/j.jamcollsurg.2008.02.011
24. Birchley G, Huxtable R, Ives J, et al. Have We Made Progress in Identifying (Surgical) Innovation? *The American Journal of Bioethics* 2019;19(6):25-27. doi: 10.1080/15265161.2019.1602181
25. Cousins S, Blencowe NS, Blazeby JMJBo. What is an invasive procedure? A definition to inform study design, evidence synthesis and research tracking. 2019;9(7):e028576.

For peer review only

26. Medical Devices Regulations 2002 (SI 2002 No 618 aaUM. [Available from: https://www.gov.uk/guidance/medical-devices-how-to-comply-with-the-legal-requirements accessed 11th January 2021. .
27. Smith JAJRmip. Semi-structured interviewing and qualitative analysis. 1995
28. Britten NJB. Qualitative research: qualitative interviews in medical research. 1995;311(6999):251-53.
29. Rubin HJ, Rubin IS. Qualitative interviewing: The art of hearing data: sage 2011.
30. Hammersley P, Atkinson M. Ethnography: principles and practice. London: Routledge 1983.
31. Charmaz K, Belgrave LJTShoirTcotc. Qualitative interviewing and grounded theory analysis. 2012;2:347-65.
32. Donovan JL, Rooshenas L, Jepson M, et al. Optimising recruitment and informed consent in randomised controlled trials: the development and implementation of the Quintet Recruitment Intervention (QRI). *Trials* 2016;17(1):283. doi: 10.1186/s13063-016-1391-4
33. Elliott D, Husbands S, Hamdy FC, et al. Understanding and Improving Recruitment to Randomised Controlled Trials: Qualitative Research Approaches. *Eur Urol* doi: 10.1016/j.eururo.2017.04.036
34. Blencowe NS, Blazeby JM, Donovan JL, et al. Novel ways to explore surgical interventions in randomised controlled trials: applying case study methodology in the operating theatre. 2015;16(1):589.
35. Blencowe NS, Skilton A, Gaunt D, et al. Protocol for developing quality assurance measures to use in surgical trials: an example from the ROMIO study. 2019;9(3):e026209.
36. Braun V, Clarke V. Using thematic analysis in psychology. *Qualitative Research in Psychology* 2006;3(2):77-101. doi: 10.1191/1478088706qp063oa
37. Seale C, Silverman DJTEJoPH. Ensuring rigour in qualitative research. 1997;7(4):379-84.
38. Jepson M, Elliott D, Conefrey C, et al. An observational study showed that explaining randomization using gambling-related metaphors and computer-agency descriptions impeded randomized clinical trial recruitment. 2018;99:75-83.
39. Kalekin-Fishman D. Review: David Silverman (2001). Interpreting Qualitative Data: Methods for Analysing Talk, Text and Interaction. *2001* 2001;2(3) doi: 10.17169/fqs-2.3.923 %J Forum Qualitative Sozialforschung / Forum: Qualitative Social Research [published Online First: 2001-09-30]
40. Blencowe NS, Mills N, Cook JA, et al. Standardizing and monitoring the delivery of surgical interventions in randomized clinical trials. 2016;103(10):1377-84. doi: 10.1002/bjs.10254
41. Blencowe N, Mills N, Donovan J, et al. Methods for standardising and monitoring surgical interventions in RCTs. 2015;16(2):1-1.

For peer review only

For peer review only - http://bmjopen.bmj.com/site/about/guidelines.xhtml
